# Exposure to aflatoxin and fumonisin in children at risk for growth impairment in rural Tanzania

**DOI:** 10.1016/j.envint.2018.03.001

**Published:** 2018-06

**Authors:** Chen Chen, Nicole J. Mitchell, Jean Gratz, Eric R. Houpt, Yunyun Gong, Patricia A. Egner, John D. Groopman, Ronald T. Riley, Jency L. Showker, Erling Svensen, Estomih R. Mduma, Crystal L. Patil, Felicia Wu

**Affiliations:** a Department of Food Science and Human Nutrition, Michigan State University, East Lansing, MI, USA; b Division of Infectious Diseases and International Health, University of Virginia, Charlottesville, VA, USA; c School of Food Science and Nutrition, University of Leeds, UK; d Department of Environmental Health Sciences, Johns Hopkins University, Bloomberg School of Public Health, Baltimore, MD, USA; e Toxicology and Mycotoxin Research Unit, National Poultry Disease Research Center, R.B. Russell Research Center, USDA-ARS, Athens, GA, USA; f Haukeland University Hospital, Bergen, Norway; g Haydom Lutheran Hospital, Manyara Region, Tanzania; h Department of Women, Children and Family Health Science, University of Illinois at Chicago, College of Nursing, Chicago, IL, USA; i Uckele Health and Nutrition, Blissfield, MI, USA; j University of Bergen, Norway

**Keywords:** Aflatoxin, Fumonisin, Dietary exposure, Biomarkers, Child growth

## Abstract

Growth impairment is a major public health issue for children in Tanzania. The question remains as to whether dietary mycotoxins play a role in compromising children's growth. We examined children's exposures to dietary aflatoxin and fumonisin and potential impacts on growth in 114 children under 36 months of age in Haydom, Tanzania. Plasma samples collected from the children at 24 months of age (N = 60) were analyzed for aflatoxin B_1_-lysine (AFB_1_-lys) adducts, and urine samples collected between 24 and 36 months of age (N = 94) were analyzed for urinary fumonisin B_1_ (UFB_1_). Anthropometric, socioeconomic, and nutritional parameters were measured and growth parameter z-scores were calculated for each child. Seventy-two percent of the children had detectable levels of AFB_1_-lys, with a mean level of 5.1 (95% CI: 3.5, 6.6) pg/mg albumin; and 80% had detectable levels of UFB_1_, with a mean of 1.3 (95% CI: 0.8, 1.8) ng/ml. This cohort had a 75% stunting rate [height-for-age z-scores (HAZ) < −2] for children at 36 months. No associations were found between aflatoxin exposures and growth impairment as measured by stunting, underweight [weight-for-age z-scores (WAZ) < −2], or wasting [weight-for-height z-scores (WHZ) < −2]. However, fumonisin exposure was negatively associated with underweight (with non-detectable samples included, *p* = 0.0285; non-detectable samples excluded, *p* = 0.005) in this cohort of children. Relatively low aflatoxin exposure at 24 months was not linked with growth impairment, while fumonisin exposure at 24–36 months based on the UFB_1_ biomarkers may contribute to the high growth impairment rate among children of Haydom, Tanzania; which may be associated with their breast feeding and weaning practices.

## Highlights

• Growth impairment is one of the key indicators of child malnutrition.
• The children (under 36 months) cohort in Haydom, Tanzania had a 75% stunting rate.
• Low aflatoxin exposure was not associated with growth impairment in this cohort.
• Fumonisin exposure linked to underweight may contribute to child growth impairment.

## 1 Introduction

Growth impairment is one of the key indicators of child malnutrition, and is an underlying cause in 2.9 million deaths in children under age 5 worldwide in 2011 (Black et al., 2013). Beyond the indication of malnutrition, childhood stunting, underweight, and wasting have been associated with increased vulnerability to infectious diseases, cognitive impairment lasting well beyond childhood, and potentially reduced adulthood achievement (Blössner and Onis, 2005; Schaible and Kaufmann, 2007; Guerrant et al., 2008; Victora et al., 2008). Most of these cases of child growth impairment occur in resource-poor regions of the world in Africa, South Asia, and Central America (Black et al., 2013).

There are an estimated 130 deaths of children under age five every day in Tanzania (URT and UNICEF, 2016). Growth impairment may play an important role in these deaths. Tanzania has the third highest rate of childhood stunting and underweight among African nations, after Ethiopia and the Democratic Republic of Congo (United Republic of Tanzania (URT) and United Nations Children's Fund (UNICEF), 2016). In 2016, the prevalence of stunting (height-for-age z-score < −2), underweight (weight-for-age z-score < −2) and wasting (weight-for-height z-scores < −2) for children under five years old was 34%, 14%, and 5%, respectively (MoHCDGEC et al., 2016). Although multiple factors are involved in child growth impairment, there has been a growing interest in understanding the role of dietary and environmental toxins in increasing the risk.

Aflatoxin is a dietary mycotoxin produced by the fungi *Aspergillus flavus* and *A. parasiticus*, which commonly infect food crops in warm climates worldwide (Kensler et al., 2011) The main sources of aflatoxin exposure in humans are maize and groundnuts, which are consumed in significant amounts by many populations worldwide; particularly in low- and middle-income nations in tropical/subtropical climates (Villers, 2014; Wu and Guclu, 2012). “Naturally occurring mixes of aflatoxins” are classified as a Group 1 human carcinogen by the International Agency for Research on Cancer (IARC, 1993). Aflatoxin exposure causes hepatocellular carcinoma (HCC), or liver cancer, which is the third-leading cause of cancer deaths worldwide. Most of these aflatoxin-related cancer cases occur in sub-Saharan Africa, China, and Southeast Asian countries where people subsist largely on cereal and cereal products (Liu and Wu, 2010). Moreover, extremely high doses of aflatoxin may cause acute aflatoxicosis in animals and humans; symptoms include internal hemorrhaging, acute liver damage, edema, and death (Khlangwiset et al., 2011). In the last two decades, interest has grown in the potential role of aflatoxin in child growth impairment and various epidemiological studies have shown associations between aflatoxin exposure and growth impairment among children in Africa and the Middle East, where maize and nuts are dietary staples (Gong et al., 2002, Gong et al., 2004; Okoth and Ohingo, 2004; Turner et al., 2003, Turner et al., 2007; Shuaib et al., 2010). However, two recent studies did not find an association between aflatoxin exposure and impaired growth of children in Nepal and Tanzania (Mitchell et al., 2017; Shirima et al., 2015).

Fumonisin, another mycotoxin, is produced by the fungi *Fusarium verticillioides* and *F. proliferatum* species, which also commonly infect maize and maize-based products in warm climates worldwide. Fumonisin B_1_, which is the most prevalent form of fumonisins, has been classified by IARC as a Group 2B possible human carcinogen (IARC, 2002). The Joint FAO/WHO Expert Committee on Food Additives (JECFA) established a provisional maximum tolerable daily intake (PMTDI) for fumonisins of 2 μg/kg bw/day on the basis of a no observed adverse effect level (NOAEL) for nephrotoxicity in male rats and an extrapolation factor of 100 (Bulder et al., 2012). Fumonisin has been shown to be associated with esophageal cancer in Asian and South African adult populations: higher fumonisin exposure from dietary sources correlated with higher number of esophageal cancer cases (Alizadeh et al., 2012; Shephard et al., 2002; Sun et al., 2007, Sun et al., 2011); and neural tube defects (NTDs) in human babies whose mothers were exposed to high level of fumonisins through consumption of maize-based food during the first trimester of pregnancy (Missmer et al., 2006).

Although dietary fumonisin intake is high among young children in rural areas of sub-Saharan African countries (Kimanya et al., 2010), only two epidemiological studies on the relationship between fumonisin exposure and child growth have been conducted. Kimanya et al. (2010) found that infants in Tanzania with relatively higher fumonisin intakes (exceeding the JECFA's PMTDI of 2 μg/kg bw/day, estimated from caregivers' dietary recall questionnaires) were significantly shorter and lighter than those whose fumonisin intakes were below the JECFA's guidance value (Kimanya et al., 2010). Shirima et al. (2015) reported that there was a negative relationship between fumonisin exposure and child growth among children from four villages in Tanzania, based on validated urinary biomarker levels of fumonisin exposure. By contrast, aflatoxin exposure did not have a significant impact independently on child growth in these cohorts; although co-exposure to these two mycotoxins was associated with impaired growth. It was concluded that fumonisin exposure is a significant risk factor in length and weight of the young children after the covariates were adjusted. However, other risk factors such as micronutrient status or exposure to infectious agents were not taken into account in these studies. The Etiology, Risk Factors, and Interactions of Enteric Infections and Malnutrition and the Consequences for Child Health and Development program (MAL-ED) is a multi-institutional project led by the Foundation of the National Institutes of Health (FNIH, 2018). It is a prospective longitudinal cohort study examining children from birth to 36 months of age, to identify risk factors compromising child health and development in eight low- to middle-income sites worldwide (MAL-ED Network Investigators, 2014). Traditionally, vitamin deficiencies, poor hygiene and lack of sanitation facilities (water scarcity and poor quality, lack of access to proper toilets), and infectious diseases (e.g. diarrhea, malaria) and dietary behaviors, including favoring boys over girls in food availability have been considered major risk factors in child growth impairment in low-income countries (Hadley et al., 2008; Katona and Katona-Apte, 2008; Levinson et al., 2004; Schmidt, 2014). However, after interventions were implemented to reduce growth impairment in some West African countries, including micronutrient supplementation and routine vaccinations, child stunting rates remain high (Ahmed et al., 2012). These results suggest the presence of other factors in stunting in these high-risk populations.

A contributing factor to poor growth may be dietary toxins. Mycotoxins are common contaminants of staple food that make up a large proportion of weaning foods of children. This study focuses on the potential role of dietary exposures to two of the most prevalent mycotoxins in maize - aflatoxin and fumonisin - on children's growth at the ages of 24 to 36 months of life in Haydom, Tanzania.

## 2 Materials and methods

### 2.1 Study design and description of study site

A detailed study design for the entire MAL-ED network of investigators is provided in the series of MAL-ED publications (MAL-ED Network Investigators, 2014). The Tanzania site within the MAL-ED network is Haydom, located in northcentral Tanzania approximately 300 km from Arusha. Haydom has a population of about 23,000 inhabitants of various ethnicities, and is a geographically diverse set of rural villages. This MAL-ED study area was chosen due to the comparatively high rates of growth impairment which is 55% among children aged 24 to 60 months according to a recent study (Psaki et al., 2014), the presence of Haydom Lutheran Hospital, and its clinics that support an efficient reproductive health system across the area (Ahmed et al., 2012; Mduma et al., 2014). In the present study, mother-infant dyads were recruited from communities within the Manyara Region of Haydom, Tanzania over a 2-year period beginning in November 2009. Determination of included study areas within the Manyara region is described in detail in Mduma et al. (2014). Location relative to Haydom Lutheran Hospital and child growth parameters were determining factors for inclusion of specific villages into the cohort. The Haydom cohort is a socioeconomically marginalized, rural community consisting primarily of subsistence farming households. Selection bias was minimized by defining a representative mother/child population and screening every mother for eligibility. Inclusion criteria included: mother aged 16 years or older, singleton pregnancy, intention to remain in the study area for at least 6 months following enrollment, and birth weight or enrollment weight of >1500 g. Exclusion criteria included diagnosis of congenital disease or severe neonatal disease (MAL-ED Network Investigators, 2014).

IRB approval was obtained from the National Health Research Ethics Committee, which is part of the National Institute for Medical Research of Tanzania, and Michigan State University. In total, plasma samples collected at 24 months of age (N = 60) were utilized for measuring aflatoxin B_1_-lysine (AFB_1_-lys) biomarker concentrations and urine samples collected between 24 and 36 months of age (N = 94) were analyzed for urinary fumonisin B_1_ (UFB_1_) concentrations. Eighteen of the participants had both a plasma and a urine sample for the AFB_1_-lys and UFB_1_ analysis at the same timepoint of 24 months.

### 2.2 AFB_1_-lys plasma biomarker analysis

AFB_1_-lys is a well-established biomarker of long-term dietary aflatoxin exposure during the past 2–3 months. Its concentrations were determined by liquid chromatography isotope dilution mass spectrometry (LC/MS) as described in Groopman et al. (2004) and McCoy et al. (2008). Briefly, plasma (200 μl) was vortexed with internal standard, 10 μl × 0.1 ng AFB_1_-D4-lys/ml, and pronase and incubated for 18 h at 37 °C. Samples were passed through solid-phase extraction (SPEs) columns and the eluent analyzed using ultra performance liquid chromatography - tandem mass spectrometer (UPLC-MS/MS). The parent ion for AFB_1_-D4-lys [(M+H)^+^, *m*/*z* 461.3] fragmented to yield a daughter ion at *m*/*z* 398.2. The AFB_1_-lys ion (*m*/*z* 457.2) fragmented to yield a daughter ion at *m*/*z* 394.1. This methodology had a limit of detection of 0.4 pg AFB_1_-lys/mg albumin and was run with quality control samples run in triplicate.

### 2.3 UFB_1_ biomarker analysis

Urinary fumonisin B_1_ (UFB_1_) has been proposed as an effective biomarker for dietary fumonisin exposure over the past 24 h, and is currently used worldwide for biomonitoring of human fumonisin exposure (van der Westhuizen et al., 2013). A significant correlation in a positive dose-dependent manner was observed between dietary fumonisin exposure and the UFB_1_ levels in human populations (Riley et al., 2015). The analytical method used for UFB_1_ was a minor modification of a method described previously (Riley et al., 2012). Briefly, urine samples (2 ml) containing 10 ng of U-[^13^C_34_]-FB_1_ (33621 Sigma-Aldrich Corp. St. Louis, MO, USA 33621) were extracted for FB_1_ with C_18_-SPE cartridges. The loaded cartridges were eluted using 2 ml of 70% acetonitrile: 30% water made to 0.1% formic acid as previously described (Riley et al., 2012). The eluates were concentrated under N_2_ at room temperature, so that the final acetonitrile-to-water concentration (based on specific gravity) was 30%–70%, and approximately 0.1% formic acid. Quantitation was accomplished by LC/MS as previously described. Normalization of UFB_1_ concentrations was described in the Supplemental material. The limit of detection for UFB_1_ is 0.01 ng/ml. The detection limits for FB_2_ and FB_3_ are similar (Riley et al., 2012).

### 2.4 Anthropometrics, socioeconomic status and dietary intake

MAL-ED trained staff members measured anthropometrics of children enrolled in the study on a monthly basis. Quality control measures included standardized techniques and instruments across study sites, and measurements were repeated on a subset of participants. Standard infant scales (SECA) were used to measure weight at the nearest 0.1 kg and length measuring board or non-stretch Teflon synthetic tape (SECA) was used to measure height at the nearest 0.1 cm, respectively. Socioeconomic status (SES) is a conceptualization of an individual's, household's or community's access to resources and can be measured using various methodologies. Prior to initiation of recruitment and consent among participants, the MAL-ED network undertook preliminary research to determine an appropriate methodology to measure SES that could be applicable in a multi-country study. Determination of the critical variables and resources allowed the MAL-ED network to formulate an index of household SES called the Water/sanitation, Assets, Maternal education, and Income (WAMI) to be applied and comparable across multiple countries (Psaki et al., 2014). Components included in the WAMI index include improved access to water and sanitation, wealth measured by a set of assets, maternal education, and monthly household income. In the present study, the methodology used for combining these components into a WAMI score was conducted according to Psaki et al. (2014).

Mothers or other adult household members were queried monthly (months 9–36 of the children's lives) to collect the children's 24-hour dietary recall data, which were used to derive food and nutrient intake information. For our analyses, we averaged monthly data points to produce estimated intakes for two age ranges: 16–24 months and 25–36 months. These data were analyzed in relation to mycotoxin biomarker concentrations. Using Haydom's food composition tables, we quantified energy, macronutrient and micronutrient intakes including vitamin A, zinc, iron, folate, protein, animal protein, and protein from milk, meat, fish, poultry, eggs, and insects (Lukmanji et al., 2008). These variables were adjusted for average total energy intake (kcal), by calculation of the residuals from an ordinary least squares regression analysis (Willett and Stampfer, 1986). The residual values were used in all statistical analyses (MAL-ED Network Investigators, 2017a).

Grain-based food items measured in the dietary recall questionnaire included rice, maize, wheat, millet, sorghum, and common beans; whereas chickpeas and mung beans are consumed at much lower frequencies. Maize makes up the main part of the children's weaning foods (Kimanya et al., 2010). These data were averaged over the timepoints 16–24 months for aflatoxin and 24–36 months for fumonisin. The data were normally distributed (goodness of fit test, *p* < 0.27) and not adjusted for statistical analysis.

### 2.5 Statistical analysis

The AFB_1_-lys data were not normally distributed, even following lognormal transformation. Therefore, all statistical analyses of AFB_1_-lys were conducted utilizing tests for non-parametric data. UFB_1_ was not normally distributed and had a significant portion of non-detectable values, therefore they were log (x + 1) transformed prior to statistical analysis. AFB_1_-lys, UFB_1_ and growth indicators were analyzed by univariate analysis with all possible confounding variables (dietary intake variables including plasma vitamin A, iron, zinc, protein and animal protein, folate, SES index, and gender). Anthropometric data (HAZ, WAZ and WHZ z-scores) were normally distributed and were not altered for statistical analysis. Wilcox-rank sum tests were used to analyze statistical differences between AFB_1_-lys or UFB_1_ concentrations and categorical data. Linear regression models were built with each growth indicator - HAZ, WAZ, and WHZ at 36 months of age - as dependent variables, and AFB_1_-lys or UFB_1_ concentrations as independent variables. A *p* value ≤ 0.05 (two-tailed) was considered statistically significant. All statistical analyses were conducted with JMP software version 13.1 (SAS Institute, Cary, NC, USA). In addition, both AFB_1_-lys and UFB_1_ concentrations were categorized by quartile, and assessed by z-scores using ANOVA followed by Tukey HSD tests to compare between quartiles and all other variables.

## 3 Results

### 3.1 Child growth and demographic variables

A total of 114 children were enrolled in the Haydom, Tanzania cohort: 59 boys and 55 girls, with mean birth height and weight of 48.8 ± 2.52 cm and 3.27 ± 0.49 kg, respectively. At 24 months, when blood samples were taken for the AFB_1_-lys analysis (n = 60; 32 boys and 28 girls), the average height and weight of the children was 79.4 ± 3.12 cm and 10.4 ± 1.31 kg at the sampling time (24 months), and averages used in association with the UFB_1_ analysis (n = 94; 49 boys and 45 girls) were 83.2 ± 4.19 cm and 11.3 ± 1.43 kg (24–36 months).

The child growth trajectories for HAZ, WAZ and WHZ scores are listed in Table 1. This cohort of children had a high incidence of stunting rate; 61% of the children had a HAZ score below −2 at 24 months when blood samples were taken for the AFB_1_-lys analysis. At 24 months, underweight incidence (WAZ < −2) rate was 17%, and 3% of the children were wasted (WHZ < −2). At 36 months, 75% of the children were stunted, 21% were underweight, and none were wasted.

**Table 1:** Statistics for growth indicators and socioeconomic status characteristics of child cohort in Haydom, Tanzania.

	Mean (95% CI)	Median	Min	Max
HAZ				
24 months	−2.45 (−2.7, −2.2)	−2.29	−4.51	−0.26
36 months	−2.63 (−2.8, −2.4)	−2.58	−5.8	−0.5
WAZ				
24 months	−1.13 (−1.38, −0.88)	−1.12	−3.89	2.02
36 months	−1.39 (−1.6, −1.1)	−1.39	−3.93	2.02
WHZ				
24 months	0.21 (−0.07, 0.49)	0.3	−2.79	2.88
36 months	0.14 (−0.04, 0.3)	0.055	−1.73	3.17
WAMI score	0.26 (0.24, 0.29)	0.27	0	0.59

**Fig. 1:**
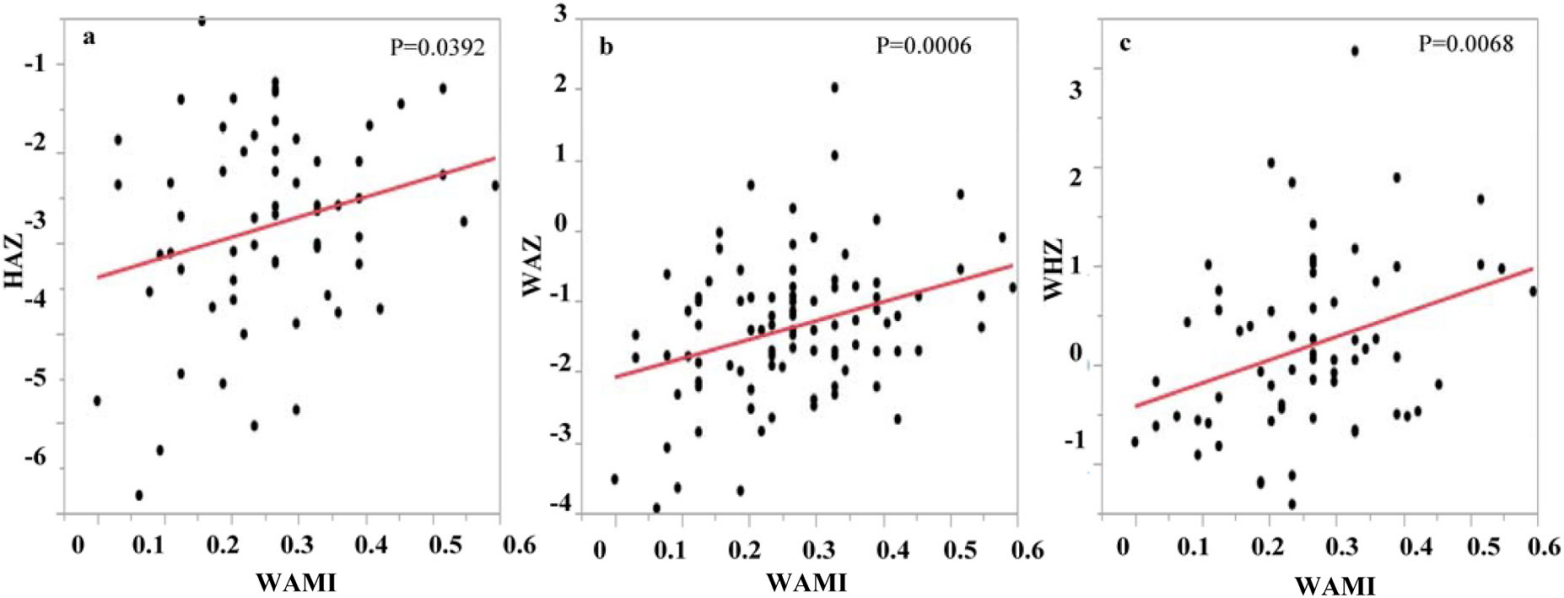
Linear regressions of WAMI index (n = 94) with HAZ (a), WAZ (b) and WHZ (c) values.

The average WAMI score, a proxy for household socioeconomic status, was 0.26 [95% CI: 0.24–0.29] (Table 1). The WAMI scores were positively associated with all three growth parameters (higher socioeconomic status was associated with improved children's growth outcomes): HAZ, *p* = 0.0392; WAZ, *p* = 0.0006; WHZ, *p* = 0.0068 (Table 3; Fig. 1a, b, c). However, the WAMI was not associated with AFB_1_-lys (*p* = 0.12) or UFB_1_ concentrations (*p* = 0.28).

### 3.2 Dietary intakes of micronutrients, proteins and grain-based foods

Table 2 shows dietary intakes of micronutrients, proteins and the number of grain-based foods for the children in Haydom, Tanzania. The mean intake of iron was 9.3 (95% CI: 8.8, 9.8) and 10.8 (95% CI: 10.4, 11.2) mg/day at age of 16–24 and 25–36 months, respectively. For zinc, the mean intake was 5.9 (95% CI: 5.6, 6.2) mg/day (16–24 months) and 6.2 (95% CI: 6.0, 6.5) mg/day (25–36 months), respectively. Intake of vitamin A in this cohort was 168 (95% CI: 147, 188) μg/day at age of 16–24 months and 140 (95% CI: 127, 154) μg/day at 25–36 months. As a main source of dietary mycotoxin exposure, the number of grain-based food items consumed daily by the children was estimated to be 5.4 (95% CI: 5.1, 5.8) at 16–24 months and 6.1 (95% CI: 5.8, 6.3) at 25–36 months. Dietary intake of folate and protein was 74.2 (95% CI: 69.4, 79.1) μg/day and 32.3 (95% CI: 30.4, 34.2) g/day at 9–24 months; and 88.5 (95% CI: 83.7, 93.4) μg/day and 31.7 (95% CI: 30.5, 32.8) g/day at 25–36 months (Table 2).

**Table 2:** Statistics of the mycotoxin exposure and dietary intakes for the children cohort (MAL-ED study) in Haydom, Tanzania.

Variable	Mean (95% CI)	Median	Min	Max
AFB_1_-lys (pg/mg albumin)	5.1 (3.5–6.6)	3.6	0.28	25.1
UFB_1_ (ng/ml urine)	1.3 (0.8–1.8)	0.4	<LOD^a^	16.6
FB_1_ dose (μg/kg bw/day)	13.8 (8.4–19.2)	4.4	0	162
Average Fe intake (mg/day)				
16–24 months	9.3 (8.8, 9.8)	9.2	5.8	13.1
25–36 months	10.8 (10.4–11.2)	11.2	5.5	14.8
Average Zn intake (mg/day)				
16–24 months	5.9 (5.6, 6.2)	5.8	3.7	8.8
25–36 months	6.2 (6.0–6.5)	6.3	3.3	8.8
Average vitamin A intake (μg retinol equivalent (RE)/day)				
16–24 months	168 (147, 188)	170	22.4	322
25–36 months	140.1 (126.5–153.6)	128	19.8	322
Average folate intake (μg/day)				
16–24 months	74.2 (69.4, 79.1)	69.5	47.5	129
25–36 months	88.5 (83.7–93.4)	84.7	34.8	145
Average protein intake (g/day)				
16–24 months	32.3 (30.4, 34.2)	31.5	19.9	51.4
25–36 months	31.7 (30.5–32.8)	31.4	19.8	45.1
Average grain intake (# items)				
16–24 months	5.4 (5.1, 5.8)	5.2	3.4	8.4
25–36 months	6.1 (5.8–6.3)	6.1	3.5	8.7

The AFB_1_-lys analysis was from serum samples collected from children at 24 months of age, and the UFB_1_ analysis was from urine samples collected from children at 24 to 36 months of age. The intake values are the averages from 16 to 24 (n = 60) and 25 to 36 months of age (n = 94), respectively.

a Limit of detection.

**Table 3:** Significance test for linear regression coefficient between HAZ, WAZ, HAZ and the WAMI index.

Variable	Regression coefficient	Standard error	t	*p*
*HAZ*				
Intercept	−3.38	0.31	−11.07	<0.0001
WAMI	2.23	1.06	2.11	0.0392^a^

*WAZ*				
Intercept	−2.08	0.22	−9.42	<0.0001
WAMI	2.66	0.75	3.54	0.0006^a^

*WHZ*				
Intercept	−0.42	0.24	−1.75	0.0849
WAMI	2.34	0.84	2.80	0.0068^a^

a Indicates there are significant associations between HAZ, WAZ, WHZ and the WAMI index.

**Table 4:** Significance test for linear regression coefficient between UFB_1_ concentrations (with the non-detects excluded and included respectively) and WAZ score.

Variable	Regression coefficient	Standard error	t	*p*
*Non-detects excluded*				
Intercept	0.88	0.26	3.34	0.0013
WAZ	−0.44	0.15	−2.87	0.0053^a^

*Non-detects included*				
Intercept	−1.19	0.13	−8.94	<0.0001
WAZ	−0.36	0.16	−2.23	0.0285^a^

a Indicates there is a negative associations between fumonisin exposure and WAZ value.

**Table 5:** ANOVA analysis with a Tukey HSD test between UFB_1_ concentrations divided by quartiles (with non-detects included) and anthropometric z-scores (HAZ, WAZ and WHZ).

UFB_1_ quartile	N	−*x* ± *s*	F	*p*
*HAZ*				
1	23	−2.30 ± 1.13	2.74	0.0482⁎
2	24	−2.73 ± 1.11		
3	24	−2.39 ± 0.83		
4	23	−3.10 ± 1.12		

*WAZ*				
1	23	−1.28 ± 0.86	3.09	0.0310⁎
2	24	−1.27 ± 1.14		
3	24	−1.11 ± 0.89		
4	23	−1.91 ± 0.94#		

*WHZ*				
1	23	0.03 ± 0.73	1.30	0.2810
2	24	0.35 ± 1.05		
3	24	0.27 ± 1.04		
4	23	−0.10 ± 0.62		

⁎ Indicates an overall significant difference.

# Indicates a significant difference between the 3rd and the 4th quartile, with *p*-value of 0.029.

In the univariate analyses, no significant associations were observed between AFB_1_-lys concentrations among children and dietary intake of average protein (*p* = 0.35), animal protein (*p* = 0.60), iron (*p* = 0.60), folate (*p* = 0.95) and vitamin A (*p* = 0.09), nor was zinc (Spearman's ρ = 0.64) intake or grain consumption frequency (Spearman's ρ = 0.40). Similarly, none of the dietary intakes of the number of grain-based food (*p* = 0.058), protein (*p* = 0.85), animal protein (*p* = 0.80), vitamin A (Spearman's ρ = 0.66), iron (Spearman's ρ = 0.66), zinc (Spearman's ρ = 0.28), folate (Spearman's ρ = 0.39) significantly correlated with UFB_1_ concentrations in children of 24–36 months in Haydom, Tanzania.

With respect to the relationship between intakes of micronutrients and child growth indicators, iron is significantly associated with improved HAZ (*p* = 0.028), and folate is significantly associated with improved WAZ (*p* = 0.025) and WHZ (*p* = 0.015).

### 3.3 Biomarker concentrations of aflatoxin and fumonisin

Statistical analysis of plasma biomarker concentrations for aflatoxin and urine biomarker concentrations for fumonisin are listed in Table 2. For this population of children, 72% had detectable AFB_1_-lys exposure at 24 months of age, with a mean level of 5.1 (95% CI: 3.5, 6.6) pg/mg albumin; 80% had detectable levels of UFB_1_ with a mean level of 1.3 (95% CI: 0.8, 1.8) ng/ml urine at 25–36 months of age. In total, 60 plasma samples were analyzed for AFB_1_-lys, 94 urine samples for UFB_1_, and 40 members of the cohort had both blood and urine samples that were analyzed for AFB_1_-lys (24 months of age) and UFB_1_ (between 24 and 36 months of age, with the mean age of 31.5 months). Of these, 30 children tested above the limit of detection for both AFB_1_-lys and UFB_1_. As seen in studies in the USA (Riley et al., 2012) and Guatemala (Torres et al., 2014), urinary FB_2_ and FB_3_ were detected at much lower levels than expected, based on what is known about their relative abundance in *F. verticillioides*-infected maize.

The daily intake of FB_1_ was estimated using individual UFB_1_ concentration, daily urine output, body weight, and the assumption that 0.5% of the FB_1_ intake is excreted in urine (Riley et al., 2012; Supplemental material). The estimated mean (13.8 μg/kg bw/day) and median (4.4 μg/kg bw/day) levels of fumonisin intake exceeded the JECFA PMTDI of 2 μg/kg bw/day. The range was 0 to 162 μg/kg bw/day (Table 2). Fifty-six out of 94 children in the cohort exceeded the PMTDI and some of the high values are close to those known to cause adverse effects in animal studies (Rotter et al., 1996).

### 3.4 Associations between aflatoxin, fumonisin, and child growth markers

**Fig. 2:**
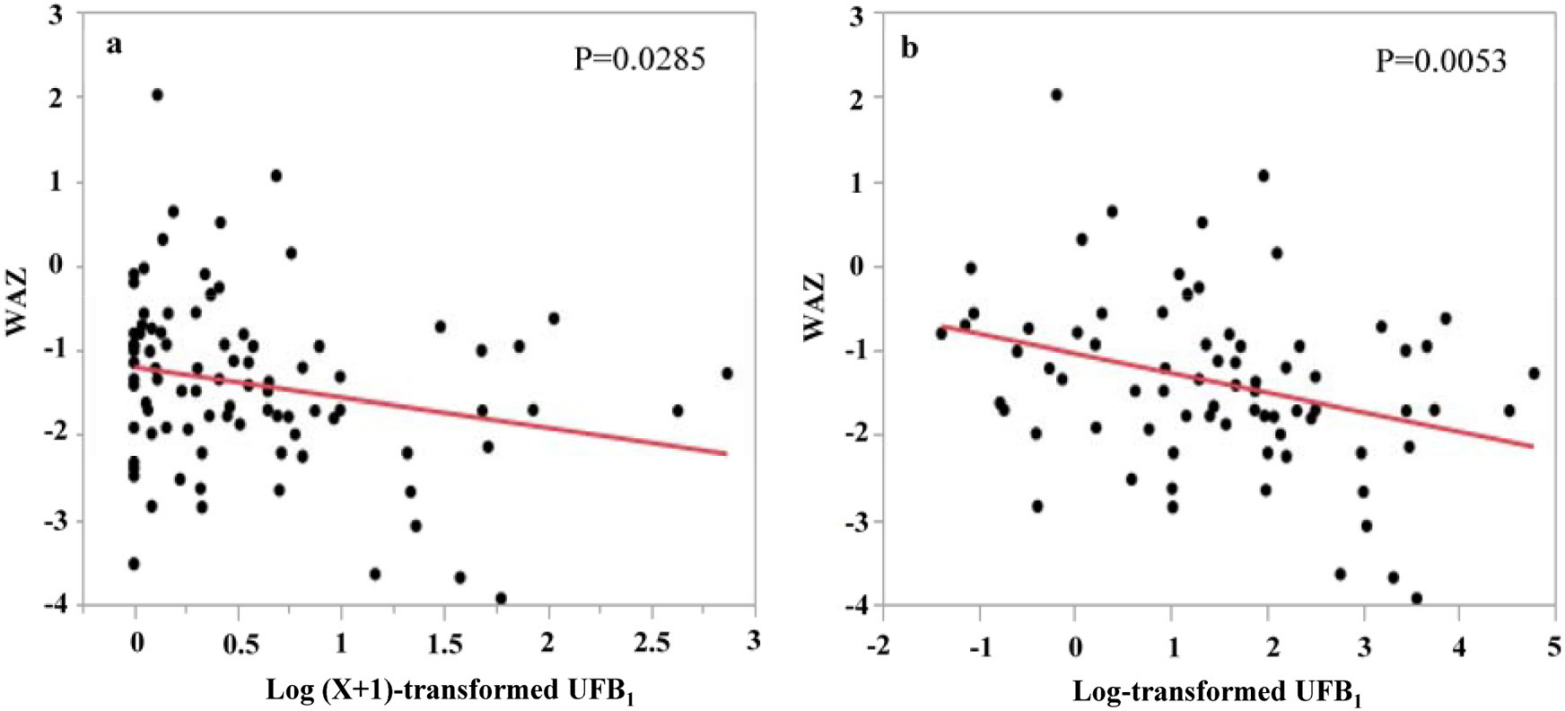
Linear regressions of WAZ and UFB_1_ concentrations, with non-detects included (a; *p*-value = 0.0285, n = 94) and non-detects excluded (b; *p*-value = 0.0053, n = 75), respectively.

Analysis of associations of log-transformed AFB_1_-lys concentrations with any of the anthropometric z-scores did not show any correlations (HAZ, *p* = 0.51; WAZ, *p* = 0.74; WHZ, *p* = 0.98). By comparison, regression analysis of UFB_1_ concentrations showed a negative association with WAZ scores (non-detectable samples excluded, *p* = 0.0053; non-detectable samples included, *p* = 0.0285) (Table 4; Fig. 2a, b), but not HAZ scores (non-detectable samples included, *p* = 0.072; non-detectable samples excluded, *p* = 0.066) or WHZ scores (non-detectable samples included, *p* = 0.27; non-detectable samples excluded, *p* = 0.060).

**Fig. 3:**
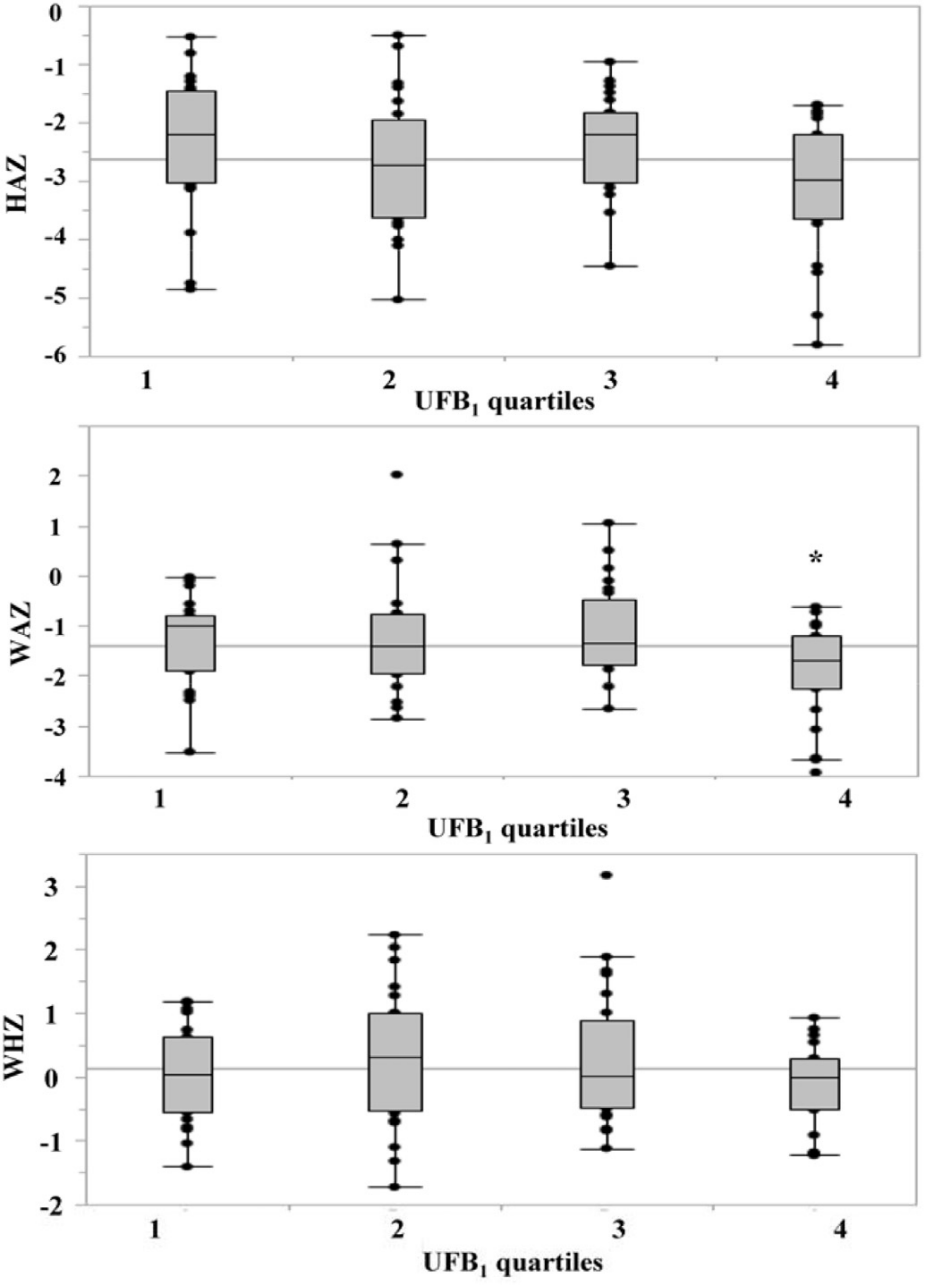
Associations of UFB_1_ concentrations (divided by quartiles, n = 23 or 24) with anthropometric z-scores (HAZ, WAZ and WHZ values). The box plots show median (solid line), the 25th and 75th percentile (upper and lower limits of the box) and 10th and the 90th percentile (error bars) for HAZ, WAZ and WHZ values. An asterisk indicates significant difference between the 3rd and the 4th quartile (*p* < 0.05).

When UFB_1_ levels were divided into quartiles (n = 23 or 24/quartile), there was a change in significance; with HAZ scores (*p* = 0.048) and WAZ scores (*p* = 0.031) being associated with biomarker concentrations of fumonisin exposure (Fig. 3), along with a significant difference between the 3rd and the 4th quartile (WAZ scores, *p* = 0.029) (Table 5). Those children in the 4th quartile (with the highest fumonisin exposure) had the lowest mean anthropometric measurements, with HAZ, WAZ, and WHZ scores of −3.1, −1.9, and −0.1, respectively (Fig. 3).

## 4 Discussion

In this cohort of children, fumonisin exposure was associated with underweight (with non-detectable samples included, *p* = 0.0285; non-detectable samples excluded, *p* = 0.0053). Similarly, Shirima et al. (2015) found a negative association between growth and fumonisin exposure in children from four other villages in Tanzania. In that study, the geometric means for fumonisin concentrations were 0.31, 0.17, and 0.57 ng/ml urine at recruitment, 6, and 12 months after recruitment, and the incidence of stunted children was 44%, 55%, and 56%, respectively; which are lower than that in the present study; where the mean UFB_1_ was 1.3 ng/ml and the incidence of stunting was 75%. Therefore, there may be a dose-dependent effect: greater fumonisin exposure resulting in greater stunting risk. In contrast to the studies in Tanzania, fumonisin exposure was low in a cohort of children in Bhaktapur, Nepal, with only one fumonisin positive sample detected among 50 urine samples, along with a lower stunting rate of 19% (Mitchell et al., 2017). Because fumonisin has been found to cause reduced weight gain, feed conversion efficiency and animal growth performance in mammal species such as swine, mice and rabbits (Abdel-Wahhab et al., 2004; Adua and Gboreb, 2015; Rotter et al., 1996), it is likely that dietary fumonisin exposure is a contributing factor to growth impairment, and a threshold of fumonisin intake is needed to affect growth.

In the present study, 72% of the children had detectable levels of AFB_1_-lys in the plasma at 24 months of age; yet no associations were found between the low aflatoxin exposures and growth impairment as measured by the children's HAZ, WAZ and WHZ values. Additionally, dietary intakes of vitamin A, iron, zinc, protein, folate, and grain consumption frequency were not correlated with aflatoxin exposure. Similarly, a recent cohort study conducted in Nepal did not find an association between aflatoxin exposure and growth impairment or growth trajectories in children's first 36 months of life (Mitchell et al., 2017). The aflatoxin concentrations are comparable, with mean AFB_1_-lys levels of 5.1 (range 0.28–25.1) and 3.62 (range 0.58–22.7) pg/mg albumin, respectively, for children in Haydom, Tanzania and Bhaktapur, Nepal.

In another longitudinal study of 166 children in Tanzania, the geometric mean for AFB_1_-alb concentrations based on enzyme-linked immunosorbent assay (ELISA) method ranged from 4.7 to 23.5 pg/mg albumin, and aflatoxin exposure was not associated with LAZ (length for height z-score) (Shirima et al., 2015). Children in that cohort (Shirima et al., 2015) had similar aflatoxin concentrations to those of the children in the present study, if we incorporate a scale factor of 2.6 to account for the different methods for aflatoxin analysis as was done in other reports (Mitchell et al., 2017). However, the scaling factor is controversial and should be used with caution. Assuming that the scale factor of 2.6 is a reasonable way to compare results using different analytical methods, then higher AFB_1_-alb concentrations were found in children of Benin and Togo, with a geometric mean concentration of 32.8 pg/mg albumin (Gong et al., 2004).

It is possible that relatively lower aflatoxin exposure levels may not affect child growth, but higher levels do. There is likely a *threshold* of aflatoxin intake, below which child growth would not be affected; and the child cohorts that showed no association between aflatoxin exposure and growth impairment may have been exposed to low aflatoxin levels overall. However, the sample size of 60 for the analysis of aflatoxin exposure in the present Haydom cohort limits the statistical power of association between AFB_1_ and child growth impairment observed. Another limitation of our study is the cross-sectional nature of the aflatoxin and fumonisin analyses, based on biospecimens at specific timepoints in the children's development.

This is partially accounted for in that the biomarker used to measure aflatoxin exposure is for long-term (past 2–3 months). Optimally, biospecimens for the mycotoxin analyses would have been available at multiple timepoints to track with growth markers; however, absent the availability, we present the exposure data that were available at specific timepoints, and observe how they correlate with child growth at those same points. In addition, it has been shown that aflatoxin biomarker levels in African children increase over the first 36 months of life, which is affected by the weaning practices (Gong et al., 2003, Gong et al., 2004). The median duration of some breastfeeding is 20 months for Tanzanian children (median duration of exclusive breastfeeding is 3 months in this population; MoHCDGEC et al., 2016). When aflatoxin exposure was analyzed at 24 months, this cohort of children likely had mixed breast feeding and weaning practices. Although their aflatoxin exposure is relatively low at the timepoint of 24 months, it could increase when they consume more maize-based foods later in their lifetime. When the fumonisin biomarker was analyzed between 24 and 36 months (mean age was 31.5 months), most children had been weaned fully for months.

The mechanisms by which mycotoxins cause growth impairment in children are still unclear. Fumonisin exerts toxic effects through its inhibition of ceramide synthase, which is a key enzyme in the biosynthesis and turnover of sphingolipids (Wang et al., 1991). Fumonisin can also affect intestinal barrier function, possibly via altered levels of complex sphingolipids in cell membranes and alterations in sphingoid base-1 phosphate signaling pathways (Bulder et al., 2012); and can mediate inflammatory responses within the gut, which may additionally potentiate or prolong infection within a damaged epithelium (Masching et al., 2016). Stunted children were found to have significantly lower serum concentrations of sphingomyelins compared with non-stunted children in rural areas of Malawi (Semba et al., 2016). Moreover, UFB_1_ levels in urine have recently been shown to positively correlate with elevated levels of sphinganine 1-phosphate and the sphinganine 1-phosphate to sphingosine 1-phosphate ratio in blood in a dose dependent manner in women in Guatemala; a result consistent with fumonisin inhibition of ceramide synthase in humans (Riley et al., 2015).

In addition to the biochemical mechanisms described above, enteric diseases have been associated with malnutrition, and malnourished children have a greater risk and increased severity of enteric illnesses (Mondal et al., 2012; Petri et al., 2008). In a vicious cycle, malnutrition contributes to the severity of the disease caused by intestinal infections, while infection in turn causes malabsorption, which predisposes individuals to further infection and malnutrition. Studies have also documented diarrheal illnesses in early childhood may account for a shortfall of growth and development of children (Guerrant et al., 2008). It is likely that the combination of both microbial and mycotoxin toxicities and their interactions within the gut and liver play a critical role in child growth impairment.

Child growth impairment rates increase with age in some countries in sub-Saharan Africa. Cessation of exclusive breastfeeding and introduction of complementary foods has been associated with an increase in aflatoxin exposure because of increased consumption of maize- and peanut-based weaning foods (Gong et al., 2003; Egal et al., 2005). In this region of Tanzania, the median duration of exclusive breastfeeding is just 38 days, and none of the infants in this study were exclusively breastfed for the recommended 6 months (Patil et al., 2015). For both adults and children, maize is the primary dietary staple. Solid or semi-solid foods including animal milk and maize porridge (mixed with milk or water) are typically the first weaning foods introduced to infants. In general, grains are the most commonly consumed food group (MoHCDGEC et al., 2016).

In the present study, the mean intake values of iron and zinc (9.3 and 5.9 mg/day, respectively at 16–24 months) are higher than recommended nutrient intakes (RNIs) which is 5.8 and 4.1 mg/day, respectively, based on the moderate bioavailability (WHO, 2004). However, accounting for lower iron and zinc bioavailability in the children's diet (comprising grains, vegetables, and animal meat at lower proportions), intake values for this children cohort are still inadequate. The recommended safe intake for vitamin A is 400 μg retinol equivalent (RE)/day, and none of the children (with the range of 22.4–322 μg RE/day) in this cohort met this requirement. In Haydom, Tanzania, about one third of children are anemic and 68% of them are vitamin A deficient at 24 months of age (MAL-ED Network Investigators, 2017b). Although the protein intake of the children met the adequacy recommended by WHO, the quality of protein may be deficient considering that relatively low proportion of the protein in their diet was from animal origin products, which can affect the nutritional status of children in this region (Ghosh, 2016). In the present study, a high prevalence of stunting was observed among children despite their high levels of protein intakes.

In a recent nutritional analysis for children aged 6–23 months in rural central Tanzania, deficiency of iron, zinc and calcium in the diets was considered as a main factor for the high prevalence of child stunting in this area, although their diets met requirements for vitamin A, protein and energy (Raymond et al., 2017). In addition, feeding practices with low nutritional quality were associated with stunting (Kulwa et al., 2015). Therefore, intervention strategies are needed to address the nutritional inadequacies and growth impairment among young children in Tanzania.

Poverty, education, socioeconomic and nutritional status, vaccinations against diseases, and dietary behaviors are traditionally considered the most important influences on childhood development. Among this cohort of children in Haydom, Tanzania, socioeconomic parameters and dietary fumonisin exposure from contaminated maize were associated with child growth outcomes.

## 5 Conclusions

The relatively low biomarker concentrations of aflatoxin at 24 months of age in this cohort of children were not linked with growth impairment, while fumonisin exposure (UFB_1_ concentrations) during 24–36 months through contaminated maize may increase the risk of underweight among children in Haydom, Tanzania; which may be associated with their breast feeding and weaning practices. Investigation of the mechanism by which fumonisin may cause growth impairment in children is recommended. Furthermore, practical interventions of fumonisin-reduction strategies are needed for sub-Saharan African populations with high fumonisin exposure through maize-based foods.

## Potential conflicts of interest

This work was supported by the Bill & Melinda Gates Foundation (all authors), the National Institute of Environmental Health Sciences of the National Institutes of Health (NJM), and the United States Department of Agriculture Agricultural Research Service (RTR, JLS).

## Appendix A Supplementary data

Supplementary material Image 1
