# Supplementary material for: Exposure to aflatoxin and fumonisin in children at risk for growth impairment in rural Tanzania
Source: Environ Int. 2018 Jun;115:29–37. doi: 10.1016/j.envint.2018.03.001 (PMC5989662; doi:10.1016/j.envint.2018.03.001)
Supplement: Supplementary file 1 — Supplementary material [file mmc1.docx]

**Supplemental Material**

**Exposure to aflatoxin and fumonisin in children at risk for growth impairment in rural Tanzania**

**Normalization of fumonisin concentrations in urine samples**

In the process of developing the method used for analysis of urinary fumonisin in Guatemalan women (Riley et al., 2012; Torres et al., 2014; Riley et al., 2015), an IRB approved study was conducted in the USA where individuals consumed tortillas and maize-based foods that were prepared from commercial products purchased at local grocery stores. Several questions were addressed concerning the best time of day to collect urine samples (AM versus PM), the stability of FB_1_ in urine samples stored frozen for up to 30 days, and the relative difference between normalizing samples to FB_1_ ng/ml versus FB_1_ ng/mg creatinine.

With regard to the difference between samples normalized per ml compared to samples normalized per mg creatinine, we found that normalizing to mg creatinine did not reduce the variability relative to that seen when normalized per ml urine (Figure S1). The urinary FB_1_ in the AM and PM samples were similar although during the period of tortilla and biscuit consumption, the PM levels were generally higher and post consumption, the PM levels were generally lower. This was apparent regardless of whether FB concentration was normalized to urinary volume or creatinine.

Figure S1. Comparison of time of day to collect urine samples (AM versus PM), and the relative difference between normalizing samples to FB_1_ ng/ml versus FB_1_ ng/mg creatinine.

**Estimation of dietary exposure to fumonisin**

The estimate for dietary exposure to fumonisin is based on the following data, assumptions and equations:

1) The relationship between the FB intake and urinary FB_1_ excretion documented in Riley et al. (2012) for adults is the same as in children between 2 and 3 years of age. Specifically, on average, 0.5% of the FB_1_ intake is excreted in the urine.

2) In countries where maize is a dietary staple, individuals consuming FB-contaminated maize on a daily basis, the level of urinary FB1 in spot urine sample are reflective of the previous 24-hour period.

3) The urine volume can be reasonably approximated using published data on daily urine output available in the literature (see Table S1 as an example). Using the data in the Table S2 below and assuming 550 ml/day on average at 24 months and 600 ml/day at 36 months the estimated urine output for each individual Tanzanian child (age 24 to 36 months) can be estimated as follows:

ml urine/day=(((months of age/36 months) × 50 ml))+550 ml/day

4) Using the estimated individual daily urine output, individual body weight, individual urinary FB_1_ (ng/ml) (Table S2), and the assumption that 0.5% of the FB_1_ intake is excreted in urine, the daily intake (µg/kg bw/day) can be estimated as follows:

µg/kg bw/day = ((ng FB_1_/ml)/0.005) × (ml urine output)/kg bw/1000

Both the estimated mean (13.8 µg/kg bw/day) and median levels (4.4 µg/kg bw/day) of intake exceeded the JECFA PMTDI of 2 µg/kg bw/day and the range is 0 to 162 µg/kg bw/day (56 out of 94 children in the cohort exceeded the PMTDI). It is critical to keep in mind that these estimates require assumptions and should be considered as a starting point, hopefully useful for designing future studies.

**Table S1. Urine output at different ages of children^a^**

| **Age** | **Output per day** |
| --- | --- |
| 0 to 48 hours | 15 to 60 ml |
| 3 to 10 days | 100 to 300 ml |
| 10 to 60 days | 250 to 450 ml |
| 2 to 12 months | 400 to 500 ml |
| [1 to 3 years](http://www.thepostnatal.com/2010/10/bread-cereals-and-potatoes-for-children/) | 500 to 600 ml |
| 3 to 5 years | 600 to 700 ml |
| 5 to 8 years | 650 to 1000 ml |
| 8 to 14 years | 800 to 1400 ml |

^a^ source: http://www.thepostnatal.com/2011/06/urine-output-at-different-ages/

**Table S2. Characteristics of children and estimated daily intake (µg/kg bw/day) of fumonisin in Haydom, Tanzania**

| **Individuals** | **Gender ^a^** | **FB_1_ (ng/ml)** | **Body weight at sampling (kg)** | **Age at sampling (months)** | **Urine output (ml)** | **FB_1_ dose (µg/kg bw/day)** |
| --- | --- | --- | --- | --- | --- | --- |
| TZ1C0001 | 2 | 0.74 | 10.9 | 30 | 592 | 8.03 |
| TZ1C0002 | 2 | 0 | 9.8 | 30.9 | 593 | 0.00 |
| TZ1C0003 | 1 | 4.54 | 11 | 30 | 592 | 48.84 |
| TZ1C0005 | 1 | 0.51 | 12.2 | 36 | 600 | 5.02 |
| TZ1C0008 | 1 | 1.18 | 11.3 | 36.1 | 600 | 12.53 |
| TZ1C0009 | 2 | 0.91 | 10.6 | 31 | 593 | 10.18 |
| TZ1C0010 | 2 | 0.12 | 11.7 | 35.9 | 600 | 1.23 |
| TZ1C0011 | 2 | 0.05 | 13.8 | 36 | 600 | 0.43 |
| TZ1C0014 | 2 | 4.37 | 11.9 | 30.9 | 593 | 43.55 |
| TZ1C0015 | 1 | 0.06 | 9.9 | 30.9 | 593 | 0.72 |
| TZ1C0016 | 1 | 0.99 | 14.5 | 30 | 592 | 8.08 |
| TZ1C0017 | 2 | 1.11 | 10.8 | 28.9 | 590 | 12.13 |
| TZ1C0018 | 1 | 0.25 | 9.8 | 28.9 | 590 | 3.01 |
| TZ1C0019 | 1 | 0.21 | 13.2 | 28.9 | 590 | 1.88 |
| TZ1C0021 | 2 | 4.9 | 8.5 | 36 | 600 | 69.18 |
| TZ1C0023 | 2 | 0.35 | 11.5 | 36 | 600 | 3.65 |
| TZ1C0024 | 2 | 1.02 | 10.6 | 29 | 590 | 11.36 |
| TZ1C0025 | 1 | 0.44 | 11.6 | 36.1 | 600 | 4.55 |
| TZ1C0026 | 2 | 0.52 | 14.8 | 36 | 600 | 4.22 |
| TZ1C0029 | 2 | 0.04 | 11.7 | 29.9 | 592 | 0.40 |
| TZ1C0031 | 2 | 2.81 | 10.2 | 29.9 | 592 | 32.59 |
| TZ1C0032 | 1 | 16.61 | 12.3 | 35.9 | 600 | 162.01 |
| TZ1C0034 | 1 | 2.21 | 9.2 | 35.9 | 600 | 28.82 |
| TZ1C0036 | 1 | 0 | 12.1 | 35.9 | 600 | 0.00 |
| TZ1C0038 | 2 | 0.35 | 12.9 | 35.9 | 600 | 3.26 |
| TZ1C0040 | 2 | 0.15 | 14.4 | 35.9 | 600 | 1.25 |
| TZ1C0041 | 1 | 6.62 | 12 | 27.9 | 589 | 64.96 |
| TZ1C0043 | 2 | 1.71 | 11.6 | 35 | 599 | 17.65 |
| TZ1C0044 | 1 | 0 | 9.7 | 23.9 | 583 | 0.00 |
| TZ1C0046 | 2 | 0 | 10.2 | 35.9 | 600 | 0.00 |
| TZ1C0049 | 2 | 1.26 | 10.5 | 36 | 600 | 14.40 |
| TZ1C0050 | 1 | 0.62 | 12 | 28.9 | 590 | 6.10 |
| TZ1C0052 | 1 | 12.87 | 11 | 26 | 586 | 137.15 |
| TZ1C0056 | 2 | 0.17 | 12.3 | 35.9 | 600 | 1.66 |
| TZ1C0057 | 1 | 0.17 | 11.4 | 36 | 600 | 1.79 |
| TZ1C0060 | 1 | 0.67 | 10.1 | 28.9 | 590 | 7.83 |
| TZ1C0060 | 1 | 2.75 | 11 | 36 | 600 | 30.00 |
| TZ1C0061 | 2 | 0.03 | 12.5 | 35.9 | 600 | 0.29 |
| TZ1C0062 | 1 | 0.92 | 10.8 | 25 | 585 | 9.96 |
| TZ1C0063 | 2 | 0.55 | 12.3 | 35.9 | 600 | 5.36 |
| TZ1C0064 | 2 | 0 | 12.5 | 35.9 | 600 | 0.00 |
| TZ1C0065 | 2 | 0.7 | 10.3 | 25 | 585 | 7.95 |
| TZ1C0068 | 1 | 0.12 | 15.3 | 24 | 583 | 0.92 |
| TZ1C0070 | 2 | 0.45 | 11 | 24.9 | 585 | 4.78 |
| TZ1C0072 | 1 | 0.39 | 11 | 36 | 600 | 4.25 |
| TZ1C0073 | 1 | 0.78 | 10.9 | 24 | 583 | 8.35 |
| TZ1C0075 | 2 | 0 | 12.3 | 35.9 | 600 | 0.00 |
| TZ1C0076 | 1 | 5.44 | 11.1 | 24.9 | 585 | 57.30 |
| TZ1C0077 | 2 | 0.39 | 8.2 | 24 | 583 | 5.55 |
| TZ1C0078 | 1 | 0 | 14 | 36 | 600 | 0.00 |
| TZ1C0079 | 2 | 2.91 | 8 | 24 | 583 | 42.44 |
| TZ1C0080 | 1 | 0.38 | 9.3 | 25.1 | 585 | 4.78 |
| TZ1C0081 | 2 | 0.11 | 9.9 | 23.9 | 583 | 1.30 |
| TZ1C0082 | 1 | 0.09 | 11.3 | 36 | 600 | 0.96 |
| TZ1C0083 | 2 | 0.05 | 10.7 | 23.9 | 583 | 0.55 |
| TZ1C0084 | 2 | 0.3 | 9.6 | 24.9 | 585 | 3.65 |
| TZ1C0085 | 2 | 0.59 | 9.4 | 24 | 583 | 7.32 |
| TZ1C0087 | 1 | 0 | 12 | 23.9 | 583 | 0.00 |
| TZ1C0088 | 1 | 0 | 10.9 | 23.9 | 583 | 0.00 |
| TZ1C0089 | 1 | 1.71 | 10 | 23.9 | 583 | 19.95 |
| TZ1C0090 | 1 | 0 | 12.1 | 35.9 | 600 | 0.00 |
| TZ1C0094 | 2 | 0.18 | 10.7 | 23.9 | 583 | 1.96 |
| TZ1C0095 | 1 | 4.39 | 10 | 24 | 583 | 51.22 |
| TZ1C0096 | 2 | 3.4 | 10.5 | 23.9 | 583 | 37.77 |
| TZ1C0098 | 2 | 0 | 12.2 | 35.9 | 600 | 0.00 |
| TZ1C0100 | 1 | 0.51 | 11.8 | 24 | 583 | 5.04 |
| TZ1C0102 | 2 | 0.09 | 9.8 | 36 | 600 | 1.10 |
| TZ1C0103 | 2 | 0 | 10.4 | 35.9 | 600 | 0.00 |
| TZ1C0105 | 1 | 0.91 | 10 | 23.9 | 583 | 10.61 |
| TZ1C0110 | 1 | 1.45 | 10.9 | 24 | 583 | 15.52 |
| TZ1C0111 | 1 | 0.08 | 12.7 | 36 | 600 | 0.76 |
| TZ1C0112 | 1 | 0.41 | 12 | 23.9 | 583 | 3.99 |
| TZ1C0113 | 1 | 1.63 | 9.9 | 24 | 583 | 19.21 |
| TZ1C0115 | 1 | 1 | 11.6 | 36 | 600 | 10.34 |
| TZ1C0118 | 1 | 0.14 | 11.1 | 24 | 583 | 1.47 |
| TZ1C0121 | 2 | 0.57 | 9.4 | 24 | 583 | 7.07 |
| TZ1C0122 | 1 | 5.89 | 10 | 23.9 | 583 | 68.70 |
| TZ1C0123 | 2 | 0.36 | 9.9 | 23.9 | 583 | 4.24 |
| TZ1C0126 | 1 | 0 | 12.7 | 36 | 600 | 0.00 |
| TZ1C0128 | 2 | 3.84 | 8.8 | 36 | 600 | 52.36 |
| TZ1C0129 | 1 | 0 | 11.4 | 36 | 600 | 0.00 |
| TZ1C0130 | 1 | 1.26 | 12.4 | 35.9 | 600 | 12.19 |
| TZ1C0133 | 1 | 0 | 10.8 | 36.2 | 600 | 0.00 |
| TZ1C0137 | 1 | 0.26 | 12 | 35.9 | 600 | 2.60 |
| TZ1C0139 | 1 | 0 | 12.1 | 36 | 600 | 0.00 |
| TZ1C0140 | 2 | 1.4 | 11.2 | 36 | 600 | 15.00 |
| TZ1C0143 | 1 | 0 | 13 | 35.9 | 600 | 0.00 |
| TZ1C0145 | 2 | 0.07 | 11.2 | 36 | 600 | 0.75 |
| TZ1C0148 | 2 | 0.09 | 12.6 | 35.9 | 600 | 0.86 |
| TZ1C0154 | 2 | 0 | 11.7 | 36 | 600 | 0.00 |
| TZ1C0156 | 1 | 1.04 | 11 | 36 | 600 | 11.35 |
| TZ1C0157 | 2 | 0 | 9 | 36 | 600 | 0.00 |
| TZ1C0158 | 2 | 0.74 | 12 | 36 | 600 | 7.40 |
| TZ1C0159 | 1 | 1.14 | 14.6 | 35.9 | 600 | 9.37 |

^a^ The number 1 and 2 indicates boy and girl, respectively.

**References**

1. Riley RT, Torres O, Showker JL, Zitomer NC, Matute J, Voss KA, et al. 2012. The kinetics of urinary fumonisin B_1_ excretion in humans consuming maize-based diets. Mol Nutr Food Res 56:1445-1455.
2. Riley RT, Torres O, Matute J, Gregory SG, Ashley-Koch AE, Showker JL, et al. 2015. Evidence for fumonisin inhibition of ceramide synthase in humans living in high exposure communities in Guatemala. Mol Nutr Food Res 59:2209-2224.
3. Torres O, Matute J, Gelineau-van Waes J, Maddox JR, Gregory SG, Ashley-Koch AE, et al. 2014. Urinary fumonisin B_1_ and estimated fumonisin intake in women from high- and low-exposure communities in Guatemala. Mol Nutr Food Res 58(5):973-83.
